# Targeted Deep Resequencing Identifies Coding Variants in the *PEAR1* Gene That Play a Role in Platelet Aggregation

**DOI:** 10.1371/journal.pone.0064179

**Published:** 2013-05-21

**Authors:** Yoonhee Kim, Bhoom Suktitipat, Lisa R. Yanek, Nauder Faraday, Alexander F. Wilson, Diane M. Becker, Lewis C. Becker, Rasika A. Mathias

**Affiliations:** 1 Genometrics Section, Inherited Disease Research Branch, National Human Genome Research Institute, National Institutes of Health, Baltimore, Maryland, United States of America; 2 The GeneSTAR Research Program, Department of Medicine, Johns Hopkins University School of Medicine, Baltimore, Maryland, United States of America; 3 Department of Anesthesiology and Critical Care Medicine, Johns Hopkins University School of Medicine, Baltimore, Maryland, United States of America; Vanderbilt University, United States of America

## Abstract

Platelet aggregation is heritable, and genome-wide association studies have detected strong associations with a common intronic variant of the platelet endothelial aggregation receptor1 (*PEAR1*) gene both in African American and European American individuals. In this study, we used a sequencing approach to identify additional exonic variants in *PEAR1* that may also determine variability in platelet aggregation in the GeneSTAR Study. A 0.3 Mb targeted region on chromosome 1q23.1 including the entire *PEAR1* gene was Sanger sequenced in 104 subjects (45% male, 49% African American, age = 52±13) selected on the basis of hyper- and hypo- aggregation across three different agonists (collagen, epinephrine, and adenosine diphosphate). Single-variant and multi-variant burden tests for association were performed. Of the 235 variants identified through sequencing, 61 were novel, and three of these were missense variants. More rare variants (MAF<5%) were noted in African Americans compared to European Americans (108 vs. 45). The common intronic GWAS-identified variant (rs12041331) demonstrated the most significant association signal in African Americans (p = 4.020×10^−4^); no association was seen for additional exonic variants in this group. In contrast, multi-variant burden tests indicated that exonic variants play a more significant role in European Americans (p = 0.0099 for the collective coding variants compared to p = 0.0565 for intronic variant rs12041331). Imputation of the individual exonic variants in the rest of the GeneSTAR European American cohort (N = 1,965) supports the results noted in the sequenced discovery sample: p = 3.56×10^−4^, 2.27×10^−7^, 5.20×10^−5^ for coding synonymous variant rs56260937 and collagen, epinephrine and adenosine diphosphate induced platelet aggregation, respectively. Sequencing approaches confirm that a common intronic variant has the strongest association with platelet aggregation in African Americans, and show that exonic variants play an additional role in platelet aggregation in European Americans.

## Introduction

The aggregation of activated platelets on ruptured or eroded atherosclerotic plaques is a critical step in the initiation of thromboses of the arterial system, which subsequently results in acute thrombosis-mediated ischemic syndromes such as myocardial infarction, stroke, and peripheral arterial occlusions [1]. The propensity of platelets to aggregate and initiate thromboses is thought to be dependent on local vascular factors, systemic factors which may change over time (such as circadian rhythms, inflammatory processes, smoking, and neuroendocrine stress), and genetic factors which modify platelet aggregability [1], [2]. Low dose aspirin (ASA, 75–81 mg daily) is given to inhibit platelet aggregation in individuals at risk for arterial thromboses and is considered standard of care for secondary prevention of coronary heart disease [3]. The Genetic Study of Atherosclerosis Risk (GeneSTAR) is a family-based study, designed to examine genetic determinants of platelet aggregation in African American and European American healthy subjects with a family history of premature coronary artery disease. In this GeneSTAR population, genetic variation was found to contribute to variability in platelet function under native conditions and after treatment with ASA as evidenced by heritability estimates [4], [5].

Candidate gene and genome-wide tests of association of agonist-induced platelet aggregability in the presence and absence of ASA identified single nucleotide polymorphisms (SNPs) in the platelet endothelial aggregation receptor (*PEAR1*, chromosome 1q23.1, RefSeq Gene: NM_001080471; Entrez Gene: 375033; Ensembl:ENSG00000187800; OMIM:610278) [6], [7]. Using fine mapping and sequence data we previously reported that a common variant (rs12041331) in intron 1 of *PEAR1* accounts most strongly for the GWAS-identified association signal between the *PEAR1* locus and platelet function phenotypes in the GeneSTAR families in both African Americans and European Americans [8]. While the strength of the association signal was stronger in African Americans, the major allele (G) at rs12041331 was associated with greater platelet aggregation in all assays across both ethnic groups under an apparent additive genetic effect. These findings were corroborated by experiments demonstrating greater platelet expression of *PEAR1* protein in relation to the G-allele at rs12041331 of the *PEAR1* gene, as well as greater luciferase expression in cells transfected with a G-allele containing reporter plasmid [8].

With the increasing availability of extensive sequence data, the hypothesis has emerged that genes first identified as harboring common GWAS-tagged variants in complex diseases may still harbor additional coding variation having collective multiple variants as well as single variant effects [9]. Given the consistent finding of a relationship between *PEAR1* and platelet aggregation, we extended the interrogation of the sequence data described above beyond the simple identification of the common variant responsible for the GWAS signal. In this study, we focus on coding variants specifically within the *PEAR1* gene to investigate the effect of common and rare exonic variants in the determination of platelet aggregation beyond that explained by the common GWAS identified intronic variant rs12041331.

## Materials and Methods

### Ethics Statement

The study was approved by the Johns Hopkins Medicine Institutional Review Board and all participants provided written informed consent. In accordance with the consents signed by the GeneSTAR subjects and the protocols approved by the Johns Hopkins Medicine Institutional Review Board, our data cannot be deposited to a public repository. Further details of our GWAS analysis are available at: http://www.ncbi.nlm.nih.gov/projects/gap/cgi-bin/study.cgi?study_id=phs000375.v1.p1. Researchers can request access to full sequence data from GeneSTAR directly through: http://www.genestarstudy.com/For-Researchers.html. Requests for this specific subset of de-identified data, for the purposes of re-analysis, can be submitted using the “Limited Access Agreement Form” (http://www.genestarstudy.com/upload/GeneSTAR%20Research%20Program%20Limited%20Access%20Agreement.pdf).”

### Participants and study protocol

The participants and protocol for GeneSTAR have been described in detail previously [4], [10]. Briefly, participants were recruited from European American (EA) and African American (AA) families with a history of premature coronary artery disease (CAD, onset <60 yrs). Healthy family members of affected probands were eligible if they were free of clinically apparent atherosclerotic disease or any other serious comorbidity. Exclusion criteria included: aspirin allergy or intolerance, anemia (hematocrit <30%), thrombocytopenia (platelet count <100,000/µL), and leukocytosis (white blood cell count >20,000/µL). Participants were given a supply of 81 mg aspirin tablets and instructed to take one pill each day for 14 days. Other than study drug, participants were prohibited from taking aspirin and other non-steroidal anti-inflammatory drugs in the 10 days prior to baseline measurements and throughout the 14 day treatment period.

A medical history, physical examination, and laboratory measurements were performed at baseline. Height and weight were measured and body mass index calculated (kg/m^2^). Venous blood was drawn after an overnight fast. Plasma levels of glucose, total and HDL cholesterol, and triglycerides were measured using standard methods (Cholestech Corporation, Hayward, CA); LDL cholesterol was calculated using the Friedewald formula. Plasma fibrinogen was measured using an automated optical clot detection device (Behring Coagulation System; Dade-Behring, Newark, DE). Hypertension, cigarette smoking, and diabetes, were defined by standard methodology as previously described 4,10.

### Platelet function

Platelet function in GeneSTAR participants was assessed at baseline and after 2 weeks of aspirin (81 mg/day). Platelet rich plasma (200,000 platelets/µl) was prepared by differential centrifugation of citrated (3.2%) blood samples. Optical aggregation was measured in a PAP-4 Aggregometer (Bio-Data Corp., Horsham, PA) after stimulating samples with collagen (2 µg/ml), adenosine diphosphate (10 µM), or epinephrine (2 µM). Peak aggregation within 5 minutes of agonist stimulation was recorded as percent (0–100%).

### Sequencing

104 GeneSTAR participants, 50 with high aggregation (26 EA, 24 AA) and 54 with low aggregation (27 EA, 27 AA), were selected for Sanger sequencing of the *PEAR1* gene. The criteria for high aggregation were: upper quartile for post-ASA aggregation to collagen (COL) 2 µg/ml, epinephrine (EPI) 2 µM, and adenosine diphosphate (ADP) 10 µM. The criteria for low aggregation were lower quartile for post-ASA aggregation to COL 2 µg/ml, EPI 2 µM, and ADP 10 µM. We stratified by race and adjusted each quantitative phenotype (COL, EPI and ADP) for age, sex, diabetes, hypertension, BMI, LDL, smoking and fibrinogen before selecting the upper and lower quartiles of aggregations. The entire *PEAR1* gene, from chromosomal 1 coordinates 156,863,523 to 156,886,226 (NCBI build 37, hg 19) with flanking regions (actual sequenced region: Chr1:156,863,190–156,892,394, hg19) was sequenced by deCODE Genetics as previously described [11]. Briefly, PCR amplifications and sequencing reactions were set up on Zymark SciClone ALH300 robotic workstations and amplified on MJR Tetrads. PCR products were verified for correct length by agarose gel electrophoresis and purified using AMPure (Agencourt Bioscience). Purified products were sequenced using an ABI PRISM Fluorescent Dye Terminator system, repurified using CleanSEQ (Agencourt), and resolved on Applied Biosystems 3730 capillary sequencers. SNP calling from primary sequence data was carried out using deCODE Genetics Sequence Miner software. *PEAR1* variants were confirmed by manual inspection of automated primary signal traces. The mean missing rate was 8.6%. We retained the 235 sequence variants identified with Sanger sequencing and all104 subjects for subsequent statistical analyses; this includes nine additional variants called in the completed dataset compared to 226 presented earlier [11]. Of 235 sequenced variants, 187 variants were located on *PEAR1* coordinates on chromosome 1from 156,863,523 to 156,886,226. Sequenced variants were annotated for variant functions (e.g., intronic, missense, synonymous), novelty (neither in dbSNP nor in 1000 Genome Project [12]), amino-acid changes and positions, and computational protein prediction using the SeattleSeq Annotation 134 (http://snp.gs.washington.edu/SeattleSeqAnnotation134, as of Oct.2012).

### Statistical analyses

Statistical tests for association were performed to look for genetic determinants of platelet hyper-aggregability comparing hyper-aggregators to hypo-aggregators within each ethnic group. In an attempt to specifically extend the single SNP common variant approach described previously [8], we performed two alternative burden tests collapsing across the exonic variants within *PEAR1* using the Combined Multivariate Collapsing (CMC) method [13], a Chi-square based approach and the Weighted Sum method (WS) [14], a score test that weights by the inverse of minor allele frequency in controls. P-values for WS were obtained through 2,000 permutations. For each of these approaches we first aggregated all exonic variants (missense and synonymous) and subsequently stratified on variant class (missense vs. synonymous). For the CMC approach only rare variants (MAF = <5%) were included and for the WS approach we looked at two strata: 1) rare exonic variants and 2) all exonic variants. We used in-house R scripts modified from publicly available R code [15], [16]. Finally, we re-evaluated the single-SNP tests for association as previously reported [8] using the Fisher's Exact Test for rare variants (MAF = <5%) and a logistic regression for common variants (MAF>5%) assuming an additive genetic model of minor alleles (i.e., number of minor alleles: 0,1,2). Covariate adjustment was made prior to sample selection, and therefore additional adjustments were not performed. Haploview [17] was used to display the linkage disequilibrium (LD) patterns and to obtain the tag SNPs accounting for LD (r^2^>0.7).

### Imputation of Sequence Variants and Association Tests in the Full GeneSTAR European American Cohort

In an attempt to replicate the findings in the EA sample using sequence data, we imputed the sequenced region using the 53 sequenced EA individuals as reference to the rest of the GeneSTAR EA cohort (N = 1,965) with prior GWAS data. We limited our imputation analysis in the EA group because there was no significant evidence of additional roles of coding variants from the burden tests in the AA group. There were 286 variants in the sequence reference set, 15 of which were also in the GWAS dataset. The reference dataset was phased using MACH v.1.0 considering 200 possible haplotypes and 100 iterations of Markov sampler. Phased haplotypes were used to impute the additional 271 variants in sequence data and the 15 variants common between sequence and GWAS data in 1,965 individuals for 498 families. We applied two cutoffs for imputation quality (Rsq) from MACH in selecting analyzable imputed genotypes: 0.7 for rare (MAF<0.05 in reference panel) variants and 0.3 for common (MAF> = 0.05 in reference panel) variants. To maximize power the discovery sequence data set was limited to samples in the extreme distributions across COL, EPI, and ADP phenotypes given the limited sample size. Here with the full cohort available for analysis of each phenotype, the full quantitative trait distributions adjusted for covariates as described above, were used for single marker association tests on imputed genotypes. Association analyses of imputed data were tested using generalized estimating equations (GEE) to account for correlation within extended families as previously described [18].

## Results

### PEAR1 sequencing data profile

**Figure 1:**
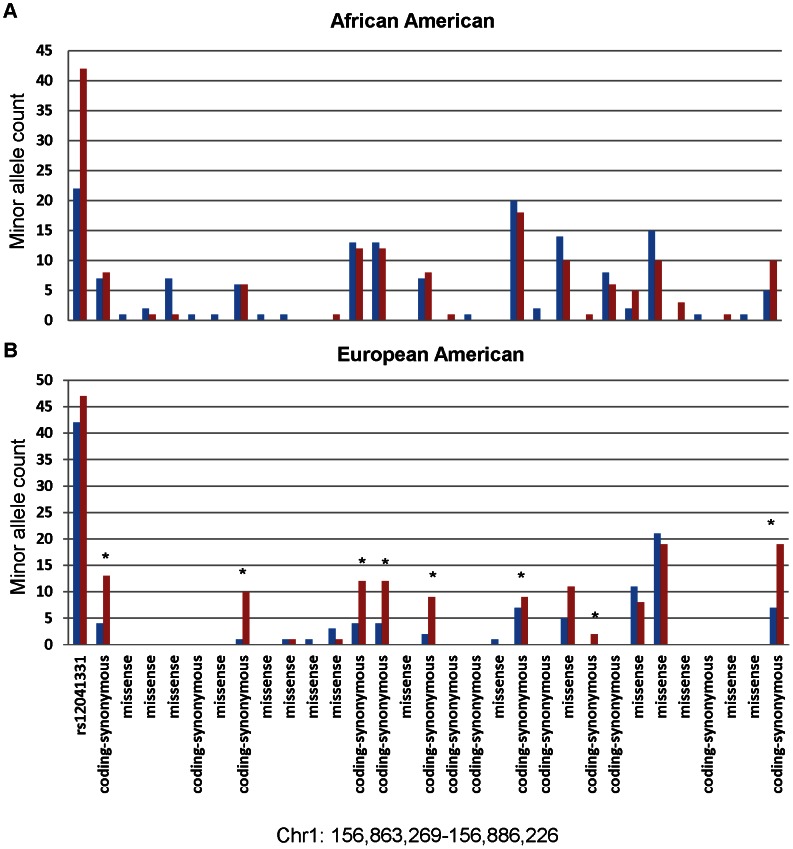
Minor allele counts of exonic variants (missense and synonymous) and the GWAS intronic variant, rs12041331. Colors of bars indicate hypo- aggregator controls (blue) and hyper- aggregator cases (red) in (A) African Americans and (B) European Americans. * indicates the synonymous variants included in the WS burden test with p = 0.0099 in European Americans (see Table 2).

A total of 235 variants were identified, of which, 61 were novel, i.e. not previously observed in either the 1000 Genomes Project subjects or in dbSNP. A total of 27(AA) and 15(EA) exonic variants were identified (missense/synonymous: 13/14 in AA and 7/8 in EA) (Figure 1, Tables 1 and 2). Of the observed missense variants, 2(15%) in AA and 1(14%) in EA were novel, while none of the synonymous variants were novel. More rare variants (MAF<5%) were noted in African Americans compared to European Americans (108 vs 45) [12].

**Table 1:** Single marker association test p values and variant annotation information for missense *PEAR1* exonic variants in African Americans (AA) and European Americans (EA).

				AA		EA		Amino Acid changes	PolyPhen2 Prediction
BP (hg19)	rs ID	Novelty^a^	Ref/Alt alleles	P-value^b^	MAF	P-value^b^	MAF		
156875126	144471706	DB	C/T	1	0.0104	NA	0	R73W	probably damaging
156875154	-	none	C/T	1	0.0326	NA	0	T82M	Benign
156877457	1952294	DB	T/C	NA	0.08	NA	0	S234P	Benign
156877782	145275734	DB	G/T	1	0.01	NA	0	G281C	probably damaging
156878039	141108373	DB	C/T	1	0.0098	NA	0	T341I	probably damaging
156878044	147639000	DB	G/A	1	0.0098	1	0.0192	D343N	possibly damaging
156878122	-	none	C/A	NA	0	1	0.0094	H369N	Benign
156878473	77795865	DB	C/T	0.4705	0.0098	0.6105	0.04	S381F	probably damaging
156879557	144560220	DB	A/G	NA	0	NA	0	N476D	Benign
156882757	41299597	DB	C/G	NA	0	1	0.0098	S802C	probably damaging
156883215	822442	DB	C/A	0.4556	0.24	0.1241	0.1538	N848K	Benign
156883493	11264581	DB	G/A	0.2158	0.0714	0.5943	0.1826	R885H	Benign
156883546	12137505	DB	A/G	0.5536	0.2659	0.8252	0.3773	N903D	Benign
156883547	78770410	DB	A/G	0.0835	0.0306	NA	0	N903S	Benign
156884486	114896181	DB	C/A	0.4489	0.0102	NA	0	P1004T	Benign
156884487	-	none	C/T	1	0.0102	NA	0	P1004L	probably damaging

a A variant novelty using catalogues in databases (DB; either dbSNP or 1000 genome database).

b Single marker association test p values using Fisher's Exact Test for rare variants (MAF = <5%) and a logistic regression for common variants (MAF>5%).

**Table 2:** Single marker association test p values and variant annotation information for synonymous *PEAR1* exonic variants in African Americans (AA) and European Americans (EA).

				AA		EA	
BP (hg19)^a^	rs ID	Novelty^b^	Ref/Alt alleles	P-value^c^	MAF	P-value^c^	MAF
156873727	12407843	DB	G/A	0.6514	0.15	0.0387	0.1603
156877477	147932853	DB	C/G	1	0.01	NA	0
*156877797 a	**77235035**	DB	C/A	0.7972	0.1395	0.0593	0.1195
156878531	11264580	DB	T/C	0.9175	0.2451	0.05	0.16
156878737	6671392	DB	T/C	0.9175	0.2451	0.05	0.16
156879580	3737224	DB	C/T	0.522	0.1562	0.0675	0.1122
156880153	143342590	DB	G/A	0.4897	0.0102	NA	0
156882590	75198597	DB	G/A	1	0.0104	NA	0
*156882996 a	**822441**	DB	C/G	0.6435	0.413	0.5469	0.1632
156883029	113502219	DB	C/T	0.4906	0.02	NA	0
*156883242 a	**55864969**	DB	G/A	0.48	0.01	0.2358	0.0188
156883263	56393520	DB	G/T	NA	0.14	NA	0
156883891	74116911	DB	T/C	1	0.0104	NA	0
*156884584 a	**56260937**	DB	C/T	0.1366	0.147	0.0107	0.2452

a Tag SNPs among 8 synonymous variants (r^2^>0.7) in European Americans.

b A variant novelty using catalogues in databases (DB; either dbSNP or 1000 genome database).

c Single marker association test p values using Fisher's Exact Test for rare variants (MAF = <5%) and a logistic regression for common variants (MAF>5%).

### Burden tests to determine the role of exonic PEAR1 variants in platelet hyper-aggregability

Table 3 shows the results of the burden tests for association in each ethnic group. In the AA group, no significant results were detected for any of the burden tests applied. In the EA group, we noted statistical significance (WS p = 0.0099) when we aggregated across all 15 exonic variants. Stratification on variant class and restriction to rare exonic variation appears to indicate that the association signal stems largely from the eight synonymous variants within *PEAR1*: the p value remains unchanged (p = 0.0099) for all eight synonymous variants in contrast to a non-significant p value when restricted to the five rare variants (p = 0.6336) or the single rare synonymous variant (p = 0.1386) using WS methods. A detailed summary of the exonic variants by hyper/hypo-aggregator status is shown in Figure 1 and Tables 1 and 2 where we see a consistent enrichment in the European American hyper-aggregators (Panel B red bars) for all the synonymous variants.

**Table 3:** Results of the burden tests (CMC, and WS) applied across the exonic variants within the PEAR1 gene: association with platelet aggregation.

		CMC	WS*	
Ancestry	Functions of aggregated variants	Rare variants^a^	Rare variants^a^	All variants^b^
		(n)^c^	(n)^c^	(n)^c^
African Americans	Missense	0.5582	0.4752	0.4455
	& Synonymous	(n = 15)	(n = 15)	(n = 27)
	Missense	0.6743	0.3168	0.495
		(n = 9)	(n = 9)	(n = 13)
	Synonymous	0.4799	0.693	0.3663
		(n = 6)	(n = 6)	(n = 14)
European Americans	Missense	0.3047	0.6336	**0.0099**
	& Synonymous	(n = 5)	(n = 5)	**(n = 15)**
	Missense	0.044	0.9009	0.594
		(n = 4)	(n = 4)	(n = 7)
	Synonymous	0.1412	0.1386	**0.0099**
		(n = 1)	(n = 1)	**(n = 8)**

* WS p values are obtained by 2,000 permutations.

a Rare variants: Analyzed using rare variants only (MAF = <5%) given the function category.

b All variants: Analyzed using rare (MAF = <5%) and common (MAF>5%) variants given the function category.

c n = number of variants used for collapsing in the analysis.

**Figure 2:**
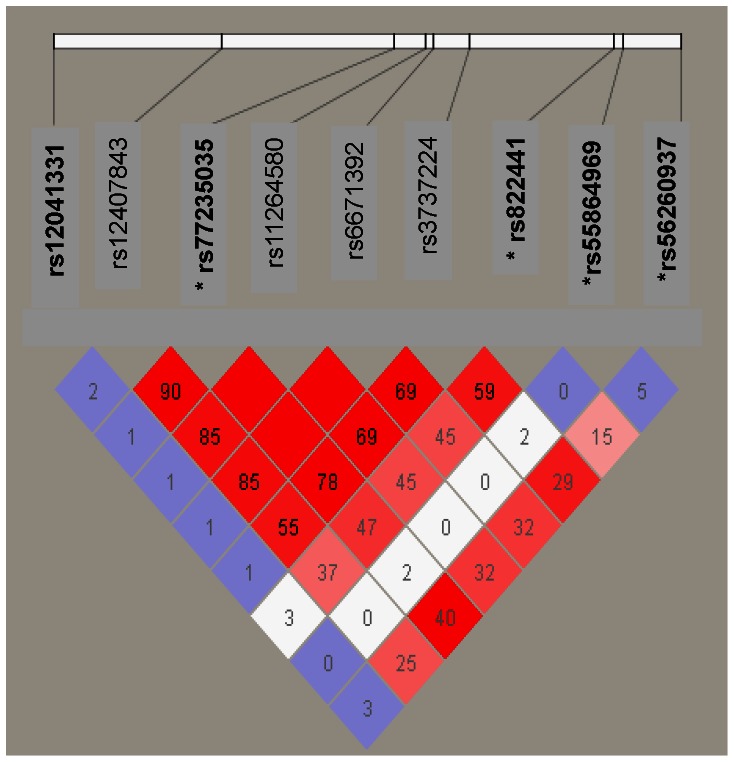
Linkage disequilibrium (LD) between the eight coding synonymous variants and rs12041331 in the European Americans. The four synonymous variants that tag the full set of eight at an r^2^>0.7 are indicated by an asterisk. The pairwise strength of LD is signified by depth of color of shaded areas (D′, white = 0, dark red = 1.0) with numbers indicating r^2^.

Figure 2 shows the pattern of LD between the 8 coding synonymous variants yielding the strongest burden test results (WS p = 0.0099, Table 3) along with the rs12041331 in European Americans. We note little to no LD between GWAS-identified rs12041331 and the exonic variants. However, there appears to be stronger LD between the 8 synonymous variants. Setting a pairwise r^2^>0.7, we determined that 4 SNPs (rs77235035, rs822441, rs55864969, and rs56260937) adequately tagged the group of 8 variants (Figure 2, Table 2), and repeating the WS test limited to these 4 variant yields a marginally stronger p-value of p = 0.0055 (in contrast to p = 0.0099 for all 8 variants).

### Summary of single-SNP tests for association stratified on ethnicity

**Figure 3:**
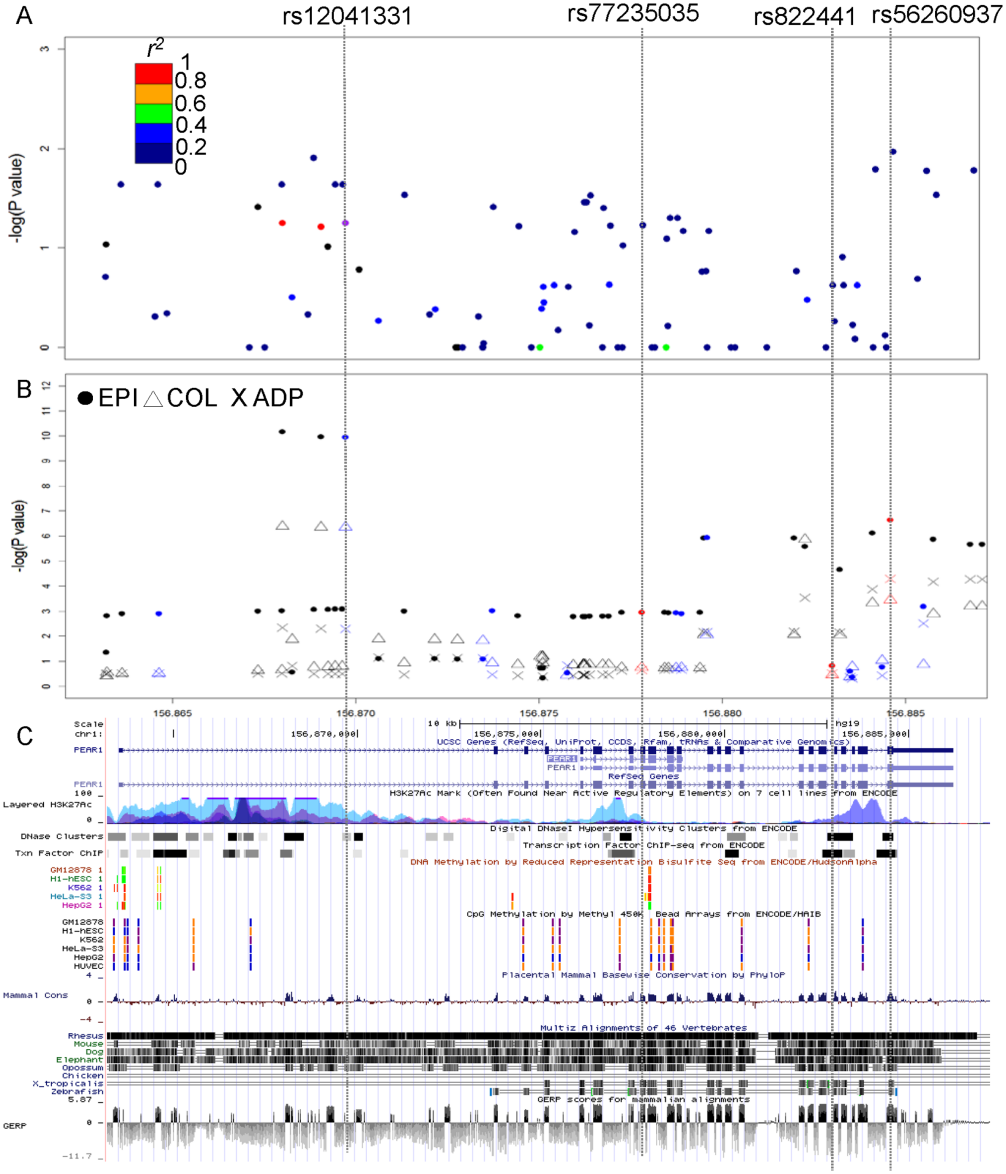
Single marker association tests for European Americans on *PEAR1* region (chr1:156,863,269–156,886,226). (A) 187 sequenced variants in the discovery set (N = 53) for hyperaggregability and (B) 55 imputed variants in the full GeneSTAR cohort (N = 1,965) for EPI (closed circle), COL (triangle), and ADP (cross) phenotypes as quantitative traits. Different colors in (A) indicate pairwise strength of LD with rs12041331 (purple). Colors in (B) indicate four tag SNPs (red), and twelve variants (blue) which are both in the GWAS and sequence datasets. The bottom panel (C) illustrates the *PEAR1* structure and regulatory regions with ENCODE regulation tracks [21]. The vertical dotted lines across all three panels indicate rs12041331, and tag synonymous SNPs rs77235035, rs822441 and rs56260937 except rs55864969 (dropped in panel B due to low imputation quality).

We previously reported in a combined analysis of the African American and European American sequenced individuals (N = 104) that the strongest signal from all of the sequence derived variants was the common intronic GWAS SNP rs12041331. An analysis stratified by the two ethnic groups yields a p value = 4.020×10^−4^ in the AA group and nominal significance in the EA group (p = 0.0565) for rs12041331. A full list of p values from single marker association tests is in the Table S1. Figure S1 illustrated the results of 235 single marker association tests for both ethnic groups. To show the effect directionality, we depicted −log10 (p values) with the direction of ‘+’ for risk effects and of ‘−’ for protective effects of each single marker. The coding variants did not yield any statistically significant results under a Bonferroni threshold (p = 0.05/235 = 0.0002) in the single-SNP tests for association (minimum p value = 0.08 for AA, 0.01 for EA) (see Tables 1 and 2, Figure 3A).

### Results of imputation of exonic variants in full GeneSTAR European American cohort

A total of 65 SNPs were successfully imputed, of which 55 SNPs were located within the *PEAR1* region (chr1:156,863,523–156,886,226). Figure 3B shows the results of the single SNP tests for association using the full GeneSTAR EA cohort and three phenotypes (COL, EPI and ADP) with full quantitative trait distributions. Imputations appear to support the association between synonymous variant rs56260937 for all three phenotypes (p = 3.56×10^−4^, 2.27×10^−7^, 5.20×10^−5^ for COL, EPI and ADP respectively). In addition, we performed conditional analysis for the rs56260937 to test the independence from rs12041331 for all three phenotypes. Association p values of rs56260937 remained significant (p = 1.44×10^−3^, 8.48×10^−7^, 1.93×10^−4^ for COL, EPI and ADP respectively) after conditioning on rs12041331 confirming independence between the two loci.

## Discussion

In our prior approach to these sequence data, we sought to validate the GWAS-identified signal at rs12041331 by combining analysis across both ethnic groups. Here, we extended our previous results to test for effects of additional exonic variants determining platelet hyper-aggregation separately in African American and European American groups. Our current approach yields no additional exonic associations in the AA group; in comparison to the peak GWAS SNP (p = 4.020×10^−4^), no burden test was significant (p = 0.3168–0.8910). In contrast, multi-variant burden tests suggest that exonic variants may play a stronger role in the EA group compared to the common GWAS variant (p = 0.0099 for the eight synonymous exonic variants in contrast to p = 0.0565 for rs12041331, the reported GWAS variant in the sequenced subset). This observation of coding synonymous effects on phenotype noted only in the European American sequenced sample may be reasonable in light of our observation that in the African American sequenced sample, rs12041331 accounts for 39% of the phenotype variance in contrast to only 16% in the European American samples, i.e. there is more residual unexplained variance in the latter once the reported GWAS intronic variant is accounted for. Ignoring the direction of allelic effects in the burden tests can lead to decreased power, so, we also implemented the C-alpha method [19] to account for this. The C-alpha test is designed for testing the variance of distribution of variants without aggregating those, which may be robust to the opposite directionalities of variant effects. We see no additional evidence for association of exonic variants with platelet hyperaggregation using the C-alpha method (p = 0.06 in the AA group and p = 0.32 in the EA group).

Synonymous variants may have a role in determining protein levels, functions and structure by 1) disruption of the spliceosome, 2) alteration in mRNA degradation, 3) control of the kinetics of translation in terms of speed and accuracy, and/or 4) removal of a ribosomal pause site, which can result in human diseases (see [20] for comprehensive reviews and details). Additionally, a recent review [20] highlighted that accumulated global understanding of the roles of synonymous variants may help direct new clinical applications and therapies. It was notable that ENCODE data indicate 3 out of the 4 tag SNPs in the final significant burden test in the EA group were located in DNaseI hypersensitivity clusters that are vulnerable to enzyme attack resulting in disruption of chromatin structures and transcription factor binding sites (Figure 3C) [21]. However, further studies are required to investigate the function of these synonymous variants and protein structure/function in the association between the *PEAR1* gene and platelet aggregation phenotypes. Of note, we limit our investigation in this study to exonic regions for identifying the direct functional variants with the emphasis on the interrogation of PEAR1 sequence variants above and beyond the intronic common GWAS-identified locus. However, as more in depth annotation becomes available, searches based on added regulatory regions may also be warranted for future studies.

In conclusion, our analysis appears to support the role of exonic synonymous variants in platelet hyper-aggregation. Imputation of the synonymous variants to the rest of the GeneSTAR European American cohort relying on the full quantitative trait distribution appears to support the role of these variants noted in the smaller discovery subset where we used phenotype extremes in sample selection. Additional research is needed to validate the imputed variants using a genotyping strategy and to identify mechanisms to examine how these synonymous coding variants cause changes in platelet aggregation. Nonetheless, our results are consistent with the emerging hypothesis that genes first identified as harboring common GWAS-tagged variants in complex diseases may harbor additional coding variation having collective multiple-variant as well as single-variant effects.

## Supporting Information

Table S1 **Single SNP association test p-values for all 235 sequenced variants including** ***PEAR1*** **region (chr1: 156,863,269–156,886,226) found in African American (AA) and European American samples (EA).**
(PDF)

Figure S1 **Single marker association test results of 235 sequenced variants.** Y-axis indicates −log_10_ (P-value) with direction of coefficients using Fisher's Exact Test for rare variants (MAF = <5%) and a logistic regression for common variants (MAF>5%) for African American (panel A) and European American (panel B) ethnic groups. Color of each bar indicates the locus functional type: 1) black: intronic/intergenic, 2) red: missense, 3) green: synonymous, 4) sky blue: 3 UTR and 5) dark blue: 5UTR (hg19). Horizontal red lines indicate Bonferroni thresholds at ±3.69 (−log_10_ (0.0002)).
(TIFF)
