# Supplementary material for: Targeted Deep Resequencing Identifies Coding Variants in the PEAR1 Gene That Play a Role in Platelet Aggregation
Source: PLoS One. 2013 May 21;8(5):e64179. doi: 10.1371/journal.pone.0064179 (PMC3660448; doi:10.1371/journal.pone.0064179)
Supplement: Table S1 — Single SNP association test p-values for all 235 sequenced variants including PEAR1 region (chr1: 156,863,269–156,886,226) found in African American (AA) and European American samples (EA). (PDF) [file pone.0064179.s001.pdf]

Supplementary information

Targeted deep re-sequencing identifies coding variants in the *PEAR1* gene that play a role in platelet aggregation

Supplementary table S1. Single SNP association test p-values for all 235 sequenced variants including PEAR1 region (chr1: 156,863,269-156,886,226) found in African American (AA) and European American samples (EA)

BP(hg19)	rsID	Ref	Alt	Function	AA		EA	
					MAF	P-value[a]	MAF	P-value[a]
156863190	3118645	G	T	intergenic	0.30	0.8772	0.20	0.1960
156863204	2935227	G	T	intergenic	0.46	0.2157	0.19	0.0928
156863532	4661071	T	C	utr-5	0.09	0.0703	0.00	NA
156863617	4661072	G	T	utr-5	0.20	0.9172	0.16	0.0232
156863967	0	A	G	intron	0.01	1.0000	0.00	NA
156864115	78616846	C	T	intron	0.03	0.5988	0.00	NA
156864195	114762366	T	C	intron	0.07	NA	0.00	NA
156864301	76164767	T	C	intron	0.05	1.0000	0.00	NA
156864441	115134729	C	T	intron	0.14	NA	0.00	NA
156864544	142135020	G	C	intron	0.00	NA	0.01	0.4898
156864616	998459	G	C	intron	0.16	0.5580	0.16	0.0232
156864688	146694129	C	T	intron	0.01	1.0000	0.00	NA
156864794	80342528	C	A	intron	0.07	NA	0.00	NA
156864856	0	G	A	intron	0.01	1.0000	0.01	0.4565
156864869	74116905	C	T	intron	0.03	0.1078	0.00	NA
156865563	77518602	G	A	intron	0.03	0.2333	0.00	NA
156865716	140869102	T	C	intron	0.01	1.0000	0.00	NA
156865924	74116906	C	T	intron	0.03	0.2326	0.00	NA
156865939	74116907	C	T	intron	0.03	0.2326	0.00	NA
156866310	0	G	A	intron	0.02	0.4400	0.00	NA
156866692	0	C	T	intron	0.01	0.4634	0.00	NA
156867098	0	G	T	intron	0.00	NA	0.01	1.0000
156867237	60998468	C	T	intron	0.03	0.2341	0.00	NA
156867286	148591218	T	C	intron	0.06	NA	0.00	NA
156867327	72698679	T	C	intron	0.11	0.2897	0.17	0.0391
156867409	141974181	C	T	intron	0.05	1.0000	0.00	NA
156867515	0	G	C	intron	0.00	NA	0.01	1.0000
156867789	114555158	G	A	intron	0.06	NA	0.00	NA
156867816	115123600	A	C	intron	0.00	0.0177	0.00	NA
156867984	6685635	C	T	intron	0.11	0.0867	0.16	0.0232
156867990	2182760	G	A	intron	0.08	0.2848	0.09	0.0565
156868254	4147300	C	A	intron	0.40	0.5404	0.24	0.3124
156868255	4147301	C	G	intron	0.40	0.5404	0.25	0.3119
156868693	0	A	T	intron	0.00	NA	0.01	0.4667

156868848	12407092	T	C	intron	0.12	0.1411	0.17	0.0125
156868891	0	G	C	intron	0.01	1.0000	0.00	NA
156869047	12566888	G	T	intron	0.48	0.0196	0.09	0.0616
156869099	149115055	C	T	intron	0.01	0.4889	0.00	NA
156869232	142243219	G	A	intron	0.01	1.0000	0.00	NA
156869237	56303495	C	T	intron	0.15	NA	0.15	0.0974
156869432	56106044	A	G	intron	0.17	0.0769	0.16	0.0232
156869630	12086222	G	C	intron	0.30	0.0223	0.16	0.0232
156869637	0	G	A	intron	0.01	0.4889	0.00	NA
**156869714**	**12041331**	**G**	**A**	**intron**	**0.29**	**0.0004**	**0.09**	**0.0565**
156870078	141619120	G	A	intron	0.00	NA	0.13	0.1647
156870615	943549	G	A	intron	0.24	0.0495	0.26	0.5415
156870769	58581379	T	G	intron	0.10	0.0913	0.00	NA
156871186	0	G	C	intron	0.01	1.0000	0.00	NA
156871296	11810069	C	G	intron	0.11	0.1941	0.00	NA
156871318	6671171	G	A	intron	0.17	0.8474	0.18	0.0294
156871995	75699653	C	T	intron	0.00	NA	0.01	0.4651
156872149	2768744	G	A	intron	0.27	0.0679	0.25	0.4120
156872164	12081638	G	T	intron	0.10	NA	0.00	NA
156872338	4661073	C	G	intron	0.04	0.2317	0.00	NA
156872589	0	A	G	intron	0.01	0.4884	0.00	NA
156872711	144019311	T	A	intron	0.00	NA	0.03	1.0000
156872767	2768745	T	C	intron	0.08	NA	0.38	0.9996
156872786	0	G	A	intron	0.01	0.4737	0.00	NA
156872895	150532926	T	G	intron	0.00	NA	0.01	1.0000
156873143	56385313	G	A	intron	0.03	0.2333	0.00	NA
156873295	78659507	G	A	intron	0.09	NA	0.00	NA
156873319	72698684	T	C	intron	0.00	NA	0.01	0.4894
156873435	145662369	G	T	intron	0.01	0.4872	0.02	1.0000
156873454	0	C	T	intron	0.00	NA	0.01	1.0000
156873463	11264579	T	C	intron	0.37	0.1833	0.26	0.9105
156873631	0	C	T	intron	0.01	1.0000	0.00	NA
156873666	58132897	C	T	intron	0.10	NA	0.00	NA
156873702	0	T	A	intron	0.01	0.4706	0.00	NA
156873727	12407843	G	A	synonymous	0.15	0.6514	0.16	0.0387
156874421	41267433	C	G	intron	0.15	0.5964	0.15	0.0608
156874765	0	G	A	intron	0.01	1.0000	0.01	1.0000
156874842	114175834	C	T	intron	0.01	0.4800	0.00	NA
156874875	0	G	A	intron	0.04	1.0000	0.00	NA
156874882	6684500	G	T	intron	0.03	0.0972	0.00	NA
156874911	73006629	T	G	intron	0.09	NA	0.00	NA
156874993	12048392	C	T	intron	0.03	1.0000	0.04	1.0000
156875016	0	A	T	intron	0.01	1.0000	0.00	NA
156875037	6676171	T	G	intron	0.39	0.4670	0.23	0.4106
156875092	4661074	G	A	intron	0.25	0.1388	0.24	0.2467

156875097	4661075	G	A	intron	0.34	0.1473	0.23	0.3529
156875107	2644590	C	A	intron	0.25	0.0642	0.23	0.3529
156875126	144471706	C	T	missense	0.01	1.0000	0.00	NA
156875154	0	C	T	missense	0.03	1.0000	0.00	NA
156875243	78814277	G	C	intron	0.00	1.0000	0.00	NA
156875360	114890085	G	A	intron	0.05	1.0000	0.00	NA
156875377	2644591	G	C	intron	0.17	0.0614	0.03	0.2358
156875482	116086124	C	T	intron	0.01	0.4706	0.05	0.6687
156875718	138955045	C	T	intron	0.02	1.0000	0.00	NA
156875764	2644592	G	A	intron	0.34	0.0494	0.18	0.2474
156875935	41267435	T	C	intron	0.34	0.9282	0.15	0.0689
156875975	76709785	T	C	intron	0.20	NA	0.00	NA
156875985	2644593	G	A	intron	0.08	NA	0.00	NA
156876206	6688345	G	A	intron	0.34	0.7434	0.14	0.0350
156876221	6688349	G	A	intron	0.34	0.8153	0.14	0.0350
156876237	6664765	C	T	intron	0.34	0.9301	0.14	0.0350
156876260	6664770	C	T	intron	0.34	0.8732	0.14	0.0350
156876274	0	G	A	intron	0.02	0.4894	0.00	NA
156876339	146185126	G	C	intron	0.01	1.0000	0.03	0.6020
156876373	6677442	A	G	intron	0.36	0.8952	0.15	0.0298
156876690	0	C	T	intron	0.00	NA	0.01	1.0000
156876729	735953	T	C	intron	0.35	0.9628	0.14	0.0397
156876877	735954	G	A	intron	0.03	0.2396	0.03	0.2353
156876906	12401524	C	A	intron	0.30	0.5713	0.14	0.0597
156877110	61813831	A	G	intron	0.03	0.2396	0.01	1.0000
156877237	115047003	C	T	intron	0.11	NA	0.00	NA
156877240	0	G	A	intron	0.01	1.0000	0.01	1.0000
156877249	6680819	A	G	intron	0.23	0.7026	0.16	0.0948
156877457	1952294	T	C	missense	0.08	NA	0.00	NA
156877477	147932853	C	G	synonymous	0.01	1.0000	0.00	NA
156877581	0	G	C	intron	0.01	1.0000	0.00	NA
156877609	0	G	A	intron	0.01	0.4800	0.00	NA
156877655	0	C	T	intron	0.01	1.0000	0.00	NA
156877782	145275734	G	T	missense	0.01	1.0000	0.00	NA
156877797	77235035	C	A	synonymous	0.14	0.7973	0.12	0.0593
156877859	0	C	A	intron	0.01	0.4792	0.00	NA
156878039	141108373	C	T	missense	0.01	1.0000	0.00	NA
156878044	147639000	G	A	missense	0.01	1.0000	0.02	1.0000
156878122	0	C	A	missense	0.00	NA	0.01	1.0000
156878420	4661076	G	A	intron	0.02	1.0000	0.04	1.0000
156878421	4661011	C	T	intron	0.02	1.0000	0.04	1.0000
156878435	11800463	C	T	intron	0.25	0.9176	0.16	0.0808
156878473	77795865	C	T	missense	0.01	0.4706	0.04	0.6105
156878531	11264580	T	C	synonymous	0.25	0.9176	0.16	0.0500
156878737	6671392	T	C	synonymous	0.25	0.9176	0.16	0.0500

156878887	11810027	G	A	intron	0.18	0.5570	0.14	0.0676
156879108	61457421	A	T	intron	0.10	NA	0.00	NA
156879155	58905602	C	T	intron	0.10	NA	0.00	NA
156879395	72698693	C	T	intron	0.23	0.8784	0.16	0.1729
156879504	41273205	A	G	intron	0.24	0.9917	0.13	0.1721
156879532	41273207	C	T	intron	0.01	0.4651	0.01	1.0000
156879557	144560220	A	G	missense	0.00	NA	0.00	NA
156879580	3737224	C	T	synonymous	0.16	0.5220	0.11	0.0675
156880153	143342590	G	A	synonymous	0.01	0.4898	0.00	NA
156880190	41273213	C	A	intron	0.00	NA	0.02	1.0000
156880288	146834392	C	G	intron	0.00	NA	0.01	1.0000
156881162	0	C	G	intron	0.00	NA	0.01	1.0000
156881200	0	T	C	intron	0.03	0.2515	0.00	NA
156881222	149259370	C	G	intron	0.04	1.0000	0.00	NA
156881382	57883920	A	G	intron	0.12	NA	0.00	NA
156881618	147227907	T	C	intron	0.04	1.0000	0.00	NA
156881959	41273215	C	T	intron	0.14	0.4643	0.13	0.1718
156881979	75966765	G	A	intron	0.03	1.0000	0.00	NA
156882261	57731889	C	T	intron	0.15	0.2527	0.07	0.3324
156882471	73006643	C	A	intron	0.06	NA	0.00	NA
156882590	75198597	G	A	synonymous	0.01	1.0000	0.00	NA
156882757	41299597	C	G	missense	0.00	NA	0.01	1.0000
156882791	12059218	C	T	intron	0.06	NA	0.00	NA
156882853	76032315	G	C	intron	0.07	NA	0.00	NA
156882950	61813832	C	T	intron	0.01	1.0000	0.03	0.2359
156882996	822441	G	C	synonymous	0.41	0.6435	0.16	0.5470
156883029	113502219	C	T	synonymous	0.02	0.4906	0.00	NA
156883084	0	G	C	intron	0.01	0.4706	0.00	NA
156883162	0	T	G	intron	0.01	0.4894	0.00	NA
156883215	822442	C	A	missense	0.24	0.4557	0.15	0.1241
156883242	55864969	G	A	synonymous	0.01	0.4800	0.02	0.2358
156883263	56393520	G	T	synonymous	0.14	NA	0.00	NA
156883346	0	C	T	intron	0.04	0.6105	0.00	NA
156883493	11264581	G	A	missense	0.07	0.2159	0.18	0.5944
156883546	12137505	A	G	missense	0.27	0.5536	0.38	0.8253
156883547	78770410	A	G	missense	0.03	0.0836	0.00	NA
156883593	112488283	G	C	intron	0.02	1.0000	0.00	NA
156883617	61813833	G	A	intron	0.04	0.1127	0.03	0.2364
156883625	78827480	T	C	intron	0.01	1.0000	0.00	NA
156883891	74116911	T	C	synonymous	0.01	1.0000	0.00	NA
156884041	146640686	C	T	intron	0.01	1.0000	0.00	NA
156884050	0	G	A	intron	0.00	NA	0.01	1.0000
156884103	703156	T	A	intron	0.50	0.2256	0.24	0.0163
156884365	749256	T	C	intron	0.08	0.3817	0.25	0.7544

156884372	75034235	G	A	intron	0.01	1.0000	0.00	NA
156884399	114364739	G	C	intron	0.00	NA	0.01	1.0000
156884486	114896181	C	A	missense	0.01	0.4490	0.00	NA
156884487	0	C	T	missense	0.01	1.0000	0.00	NA
156884584	56260937	C	T	synonymous	0.15	0.1366	0.25	0.0108
156884714	148183284	G	A	utr-3	0.06	NA	0.00	NA
156884759	55739780	T	C	utr-3	0.09	0.0787	0.00	NA
156884958	113956837	T	C	utr-3	0.07	NA	0.00	NA
156885010	0	A	C	utr-3	0.01	0.4706	0.00	NA
156885070	0	C	A	utr-3	0.01	0.4706	0.00	NA
156885156	56955334	T	C	utr-3	0.03	0.0972	0.00	NA
156885255	41273217	G	A	utr-3	0.02	0.2065	0.02	0.2048
156885400	0	C	T	utr-3	0.01	1.0000	0.00	NA
156885491	4661012	T	G	utr-3	0.30	0.0170	0.32	0.0169
156885699	0	T	G	utr-3	0.01	0.4706	0.00	NA
156885767	12139641	A	G	utr-3	0.13	0.2786	0.25	0.0294
156885851	0	A	C	utr-3	0.01	0.4800	0.00	NA
156885929	11264582	A	G	utr-3	0.07	NA	0.00	NA
156886068	0	T	C	utr-3	0.01	0.4600	0.00	NA
156886308	0	A	G	intergenic	0.01	0.4889	0.00	NA
156886354	115224276	G	A	intergenic	0.01	0.4884	0.00	NA
156886582	143644805	G	T	intergenic	0.01	1.0000	0.00	NA
156886633	147185059	A	G	intergenic	0.07	NA	0.00	NA
156886777	61546108	G	A	intergenic	0.14	0.2062	0.23	0.0168
156887101	2768746	A	G	intergenic	0.46	0.0586	0.25	0.0099
156887164	2644595	C	T	intergenic	0.05	NA	0.00	NA
156887172	149312817	G	A	intergenic	0.06	NA	0.00	NA
156887224	0	A	G	intergenic	0.01	0.4800	0.00	NA
156887312	111806293	T	C	intergenic	0.07	NA	0.00	NA
156887425	0	T	C	intergenic	0.01	0.4490	0.00	NA
156887497	148728990	C	T	intergenic	0.13	NA	0.00	NA
156887500	0	T	C	intergenic	0.01	0.4468	0.00	NA
156887580	2986717	C	T	intergenic	0.46	0.0534	0.24	0.0110
156887970	0	G	A	intergenic	0.00	NA	0.01	0.4898
156888030	77024634	G	A	intergenic	0.07	0.1237	0.00	NA
156888145	0	C	G	intergenic	0.00	NA	0.01	1.0000
156888274	0	A	T	intergenic	0.01	0.4694	0.00	NA
156888674	113179644	C	T	intergenic	0.06	NA	0.00	NA
156888690	113970934	A	G	intergenic	0.14	0.1846	0.25	0.0126
156888739	0	A	C	intergenic	0.00	NA	0.01	1.0000
156889058	114535285	C	G	intergenic	0.01	0.4600	0.00	NA
156889218	60465657	T	C	intergenic	0.08	NA	0.00	NA
156889352	0	A	C	intergenic	0.01	0.4706	0.00	NA
156889398	0	T	C	intergenic	0.00	NA	0.00	NA
156889491	0	G	A	intergenic	0.01	0.4898	0.00	NA
156889686	0	G	A	intergenic	0.01	0.4706	0.00	NA

156889930	0	G	T	intergenic	0.01	0.4898	0.00	NA
156889961	0	C	G	intergenic	0.02	1.0000	0.00	NA
156890110	142013683	C	T	intergenic	0.06	NA	0.00	NA
156890372	0	C	A	intergenic	0.02	0.2165	0.01	0.4906
156890394	0	C	T	intergenic	0.01	1.0000	0.00	NA
156890501	141256165	C	T	intergenic	0.00	NA	0.02	0.2262
156890672	41273221	T	C	intergenic	0.15	0.1366	0.24	0.0211
156890854	41273223	C	G	intron	0.03	1.0000	0.06	NA
156891004	0	T	C	intron	0.03	1.0000	0.01	1.0000
156891005	0	C	A	intron	0.03	1.0000	0.01	1.0000
156891138	115756800	C	T	intron	0.02	0.4976	0.00	NA
156891291	73006661	G	A	intron	0.03	0.1836	0.00	NA
156891554	74116913	C	A	intron	0.04	0.3112	0.00	NA
156891610	12143768	G	A	intron	0.15	NA	0.16	NA
156891688	0	G	C	intron	0.01	0.4348	0.00	NA
156891712	0	G	A	intron	0.00	NA	0.01	1.0000
156891906	55820829	G	A	intron	0.02	0.1758	0.03	0.6104
156892041	115092323	G	A	intron	0.01	1.0000	0.00	NA
156892068	0	C	G	intron	0.01	0.4490	0.00	NA
156892176	115390650	A	G	intron	0.09	NA	0.00	NA
156892394	822436	T	C	intron	0.42	0.1483	0.22	0.0295

a. Single marker association test p values using Fisher's Exact Test for rare variants (MAF =< 5%) and a logistic regression for common variants (MAF > 5%).
