# Supplementary figures and images for: Targeted Deep Resequencing Identifies Coding Variants in the PEAR1 Gene That Play a Role in Platelet Aggregation
Source: PLoS One. 2013 May 21;8(5):e64179. doi: 10.1371/journal.pone.0064179 (PMC3660448; doi:10.1371/journal.pone.0064179)

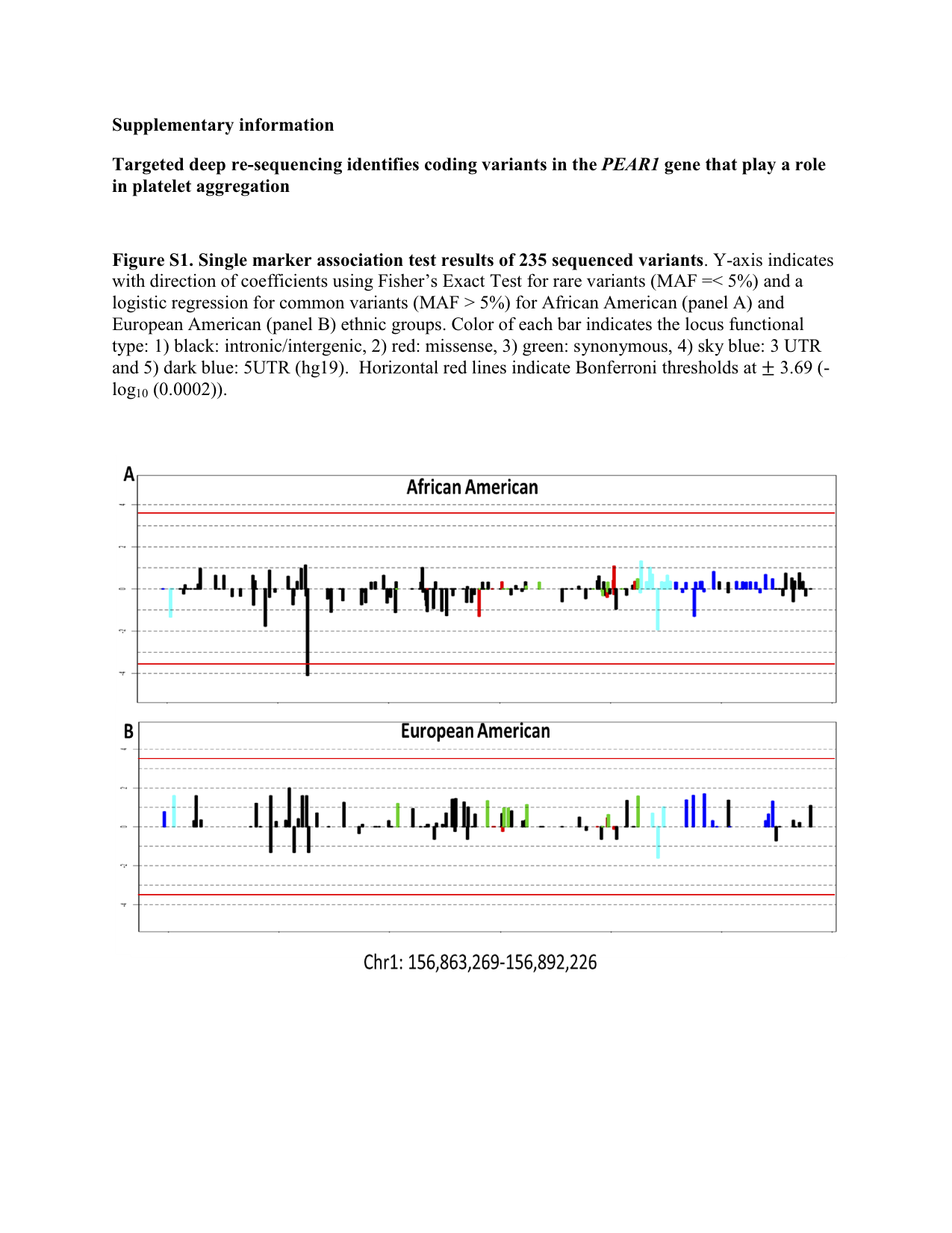

Supplement: Figure S1 — Single marker association test results of 235 sequenced variants. Y-axis indicates −log10 (P-value) with direction of coefficients using Fisher's Exact Test for rare variants (MAF = <5%) and a logistic regression for common variants (MAF>5%) for African American (panel A) and European American (panel B) ethnic groups. Color of each bar indicates the locus functional type: 1) black: intronic/intergenic, 2) red: missense, 3) green: synonymous, 4) sky blue: 3 UTR and 5) dark blue: 5UTR (hg19). Horizontal red lines indicate Bonferroni thresholds at ±3.69 (−log10 (0.0002)). (TIFF) [file pone.0064179.s002.tiff]
